# From Monogenic Variants to Polygenic Risk Scores in Inherited Cardiomyopathies: Clinical Utility, Ancestry Gaps, and Future Implementation in Arab Populations

**DOI:** 10.3390/genes17101312

**Published:** 2026-10-21

**Authors:** Sara Abou Al-Saud

**Affiliations:** Cardiac Sciences Department, College of Medicine, King Saud University, P.O. Box 7805, Riyadh 11472, Saudi Arabia; saboualsaud@ksu.edu.sa

**Keywords:** inherited cardiomyopathy, hypertrophic cardiomyopathy, dilated cardiomyopathy, arrhythmogenic cardiomyopathy, restrictive cardiomyopathy, polygenic risk score, genome-wide association study, Arab populations, Middle East, precision medicine, cardiovascular genetics

## Abstract

Inherited cardiomyopathies are major causes of heart failure, malignant ventricular arrhythmia, and sudden cardiac death, and they require lifelong surveillance of affected individuals and clinically at-risk relatives. Their clinical genetics has traditionally been organised around rare monogenic pathogenic or likely pathogenic variants, especially in sarcomeric hypertrophic cardiomyopathy (HCM), titin-related and other sarcomeric forms of dilated cardiomyopathy (DCM), nuclear-envelope DCM, and desmosomal arrhythmogenic cardiomyopathy (ACM). This model remains indispensable, but it is incomplete: it does not fully explain genotype-negative disease, variable penetrance, age of onset, disease severity, or discordant phenotypes within the same family. Polygenic risk scores (PRS), which aggregate the weighted effects of common variants, offer a second layer of inherited susceptibility that may refine the interpretation of rare variants and borderline phenotypes. Current evidence is strongest for HCM, increasingly important for DCM, and still weak for ACM and restrictive cardiomyopathy (RCM). The central limitation is equity and transferability. Most cardiomyopathy PRS discovery, calibration, and validation have been performed in European-ancestry datasets. At the same time, Arab and Middle Eastern populations remain underrepresented in GWAS, reference allele-frequency databases, longitudinal cardiac imaging resources, and clinical utility studies. Direct importation of European PRS into Arab cardiovascular genetics would therefore risk miscalibration, variant misclassification, false reassurance, overdiagnosis, and inappropriate counseling. This critical review evaluates PRS evidence across HCM, DCM, ACM, RCM, and related inherited cardiac diseases; explains why PRS is not a diagnostic test or replacement for monogenic testing; and proposes a phased Arab implementation framework based on local discovery cohorts, whole-genome sequencing, ancestry-aware calibration, family-based penetrance studies, harmonized imaging phenotypes, prospective outcome tracking, genetic counseling pathways, and governance safeguards. The future is not monogenic versus polygenic risk; it is integrated genomic risk, implemented only when clinically validated and equitable.

## 1. Introduction

Inherited cardiomyopathies are not simply genetic diagnoses; they are clinical syndromes that affect patients, relatives, surveillance programs, and decisions about exercise, pregnancy, device therapy, and heart failure prevention. HCM, DCM, ACM, restrictive cardiomyopathy, and left ventricular noncompaction can present with heart failure, atrial and ventricular arrhythmias, thromboembolism, sudden cardiac death, or an apparently silent phenotype discovered through family screening. Representative OMIM phenotype entries include HCM1 (OMIM #192600), dilated cardiomyopathy (OMIM #115200), arrhythmogenic right ventricular dysplasia/cardiomyopathy 1 (OMIM #107970), and RCM1 (OMIM #115210); each category is genetically heterogeneous and includes additional gene-specific OMIM entries. Contemporary guidelines place genetic testing, family history, electrocardiography, echocardiography, and cardiac magnetic resonance at the centre of evaluation. Still, they also make clear that phenotype, penetrance, and prognosis cannot be read directly from a single variant result [1,2].

The traditional clinical genetics workflow has been monogenic: diagnose the phenotype, test a panel of cardiomyopathy genes, classify rare variants as pathogenic, likely pathogenic, uncertain, likely benign, or benign, then use pathogenic and likely pathogenic findings for cascade testing. This remains essential. In HCM, the clinical pathway is built around sarcomeric disease, family screening, genotype-positive/phenotype-negative relatives, and sudden death risk assessment [3]. In broader cardiovascular genetics, expert consensus supports phenotype-driven testing, careful pre-test counseling, segregation analysis where possible, and regular reinterpretation of uncertain variants [4]. Ventricular arrhythmia guidelines similarly emphasize gene-specific risk in conditions such as *LMNA*-related DCM and desmosomal ACM but do not treat genetic status as a single deterministic variable [5].

The weakness of the purely monogenic model is obvious in daily practice. A pathogenic sarcomere variant does not reliably predict whether a relative will develop left ventricular hypertrophy at 18 or remain phenotype-negative at 60. A *TTN* truncating variant can be silent in one person and associated with severe DCM in another, especially when alcohol exposure, myocarditis, pregnancy, chemotherapy, hypertension, or cardiometabolic stress are present. A desmosomal variant may be expressed primarily as right-dominant ACM, left-dominant ACM, DCM overlap, or arrhythmia before structural disease. Many patients remain genotype-negative despite convincing disease, and many others receive variants of uncertain significance (VUS) that cannot responsibly drive cascade testing.

This is where PRS becomes scientifically interesting. A PRS estimates inherited susceptibility by aggregating many common variants across the genome. It does not identify a causal mutation in a family. Instead, it quantifies a background liability that may influence whether rare variant carriers express disease, whether genotype-negative individuals have polygenic or multifactorial disease, and whether borderline imaging findings represent early cardiomyopathy or secondary remodeling. The critical question is not whether PRS is statistically associated with cardiomyopathy. The critical question is whether PRS adds clinically meaningful information beyond monogenic testing, family history, ECG, imaging, established risk calculators, and modifiable exposures.

The answer is uneven. HCM now has the clearest PRS evidence because common variants, modifiable traits, and sarcomere status have been studied together. DCM is catching up through large GWAS and imaging-genetics studies, but implementation is more challenging because it is etiologically heterogeneous. ACM remains a rare-variant and phenotype-driven disease model; PRS is a research priority rather than a clinical tool. The ancestry problem cuts across all three. A PRS derived from European discovery cohorts may lose accuracy in Arab populations because allele frequencies, linkage disequilibrium, imputation performance, environmental exposures, family structures, and local founder effects differ. Arab populations are also genetically heterogeneous; Gulf, Levantine, North African, Arabian Peninsula, and diaspora communities should not be collapsed into a single undifferentiated category.

This review addresses four questions: how cardiomyopathy genetics has moved beyond a purely monogenic model; what current evidence supports PRS in HCM, DCM, ACM, and related inherited cardiac diseases; why ancestry bias limits PRS transferability; and what would be required to implement PRS responsibly in Arab populations. The review is deliberately critical. It treats PRS as a promising but unproven clinical layer, not as a substitute for careful phenotyping or validated monogenic testing.

### Review Methodology

This article uses a structured narrative-review approach. PubMed/MEDLINE and the Polygenic Score Catalog were searched from database inception through 1 August 2026, supplemented by backward and forward citation checking of major guidelines, gene-curation papers, GWAS, and PRS development and validation studies. Search concepts combined cardiomyopathy terms (hypertrophic, dilated, arrhythmogenic, restrictive, inherited cardiomyopathy) with genetic terms (monogenic, rare variant, genome-wide association study, polygenic score, polygenic risk score, penetrance, ancestry, imputation, validation) and population terms (Arab, Middle Eastern, Gulf, Levantine, and North African). Records were screened first by title and abstract and then by full text. Eligible publications were human studies addressing inherited cardiomyopathy genetics, disease-specific GWAS or PRS, PRS methodology or validation, ancestry transferability, or implementation in Arab and Middle Eastern populations.

Priority was given to contemporary guidelines, definitive or strong gene-disease curation, large GWAS, disease-specific PRS development or validation, and studies addressing ancestry transferability. Non-human studies, duplicate reports of the same analysis, and reports without relevant genetic or phenotypic data were excluded from the evidence summaries. Case reports were not used to estimate PRS performance, although selected pedigrees were retained when they directly illustrated gene-specific phenotypic heterogeneity or founder effects. For PRS studies, data extraction focused on discovery sample size and ancestry, score-construction method, number of variants, genotype quality control and imputation resources, linkage-disequilibrium reference resources, effect-size weighting, target and validation cohorts, OR or HR per standard deviation, discrimination (AUROC or C-statistic where reported), calibration, and external validation in non-European populations. An item not reported in the main article, supplement, or score metadata is labeled as not reported rather than inferred [6,7]. Because the purpose is critical synthesis rather than pooled effect estimation, the review was not prospectively registered, no formal risk-of-bias instrument was applied, and no meta-analytic summary measure was calculated.

## 2. Monogenic Genetics of Inherited Cardiomyopathies

### 2.1. Traditional Monogenic Model

Most inherited cardiomyopathy care still begins with a rare-variant framework. Many familial forms follow autosomal dominant inheritance, although recessive, X-linked, mitochondrial, and syndromic forms also occur. The practical vocabulary is penetrance, expressivity, segregation, de novo occurrence, genotype-positive/phenotype-negative status, and variant classification. Pathogenic and likely pathogenic variants can be used for cascade testing, but VUS should not be used to declare relatives affected or unaffected. The model works best when a well-established gene, a convincing phenotype, a rare damaging variant, and segregation within a family all point in the same direction.

The model fails when one of these elements is weak. Gene panels have expanded faster than evidence for the validity of gene-disease associations. Some panels include genes with disputed or limited evidence, which inflate VUS counts and create false precision. Even for strong genes, penetrance is age-dependent and exposure-dependent. A single family may contain early-onset severe disease, late-onset mild disease, and unaffected carriers. The clinical implication is blunt: monogenic testing is central, but it is not a complete risk model.

### 2.2. HCM Monogenic Architecture

HCM is the strongest example of a sarcomere-centred cardiomyopathy. Core genes include *MYH7*, *MYBPC3*, *TNNT2*, *TNNI3*, *TPM1*, *ACTC1*, *MYL2*, and *MYL3*. Contemporary reviews estimate that sarcomere genes explain approximately 30–40% of clinically diagnosed HCM, with a higher yield in familial, early-onset, or severe cases [8]. A systematic review of HCM genetic testing found meaningful diagnostic and clinical utility but also emphasized heterogeneity in study design, testing strategy, and outcome reporting [9].

The HCM genotype cannot be interpreted without the phenotype. Sarcomere-positive HCM often has an earlier onset and greater familial clustering, whereas sarcomere-negative HCM includes polygenic susceptibility, hypertension-associated remodeling, obesity-related remodeling, metabolic traits, and phenocopies. A 2024 penetrance meta-analysis showed why family counseling is difficult: penetrance depends on age, gene, variant class, ascertainment, and clinical setting rather than a fixed Mendelian probability [10]. Low-penetrance sarcomere variants further blur the boundary between monogenic and polygenic models; in a 2025 analysis, they were penetrant in only 2–12% of carriers but contributed additive pathogenic risk when combined with other risk layers [11].

Real-world HCM cohorts also show the unresolved burden of genotype-negative and uncertain results. In a 2025 HCM centre cohort of 827 patients, 754 underwent genotyping; 202 were genotype-positive, 163 carried a VUS, and 389 were genotype-negative. Genotype-positive patients had a higher risk of adverse outcomes than genotype-negative patients, with a reported composite-outcome hazard ratio of 1.84 (*p* < 0.001). Still, more than half remained genotype-negative, and more than one-fifth carried VUS [12]. That distribution is precisely why PRS has become attractive in HCM: the missing risk is not trivial.

### 2.3. DCM Monogenic Architecture

DCM is genetically broader and clinically more heterogeneous than HCM. *TTN* is the major DCM-associated gene in contemporary cohorts; it encodes titin, a giant sarcomeric protein, and truncating variants are among the most frequent monogenic findings. Other major genes include *LMNA*, *DSP*, *FLNC*, *RBM20*, *BAG3*, *PLN*, *DES*, *MYH7*, and *TNNT2*. Accordingly, DCM should not be framed primarily as a nuclear-envelope cardiomyopathy: its architecture spans sarcomeric, nuclear-envelope, desmosomal, cytoskeletal, calcium-handling, and syndromic mechanisms. Evidence-based curation has shown that not all historically reported *DCM* genes are equally valid [13]. This matters because adding weak genes to panels increases false-positive interpretation without improving patient care.

A major 2020 re-evaluation of monogenic DCM tested the strength of gene associations using large case and reference datasets, including 2538 DCM patients, 912 healthy controls, and 60,706 ExAC reference individuals [14]. The key message is not that DCM is non-genetic. Rather, many historical gene-disease claims require stricter evidence. More recent work comparing African- and European-ancestry DCM patients demonstrated how ancestry affects apparent yield: actionable variant frequencies differed substantially, with 25.5% in European-ancestry individuals versus 8.2% in African-ancestry individuals and an odds ratio of 0.25 for African versus European ancestry [15]. That finding is directly relevant to Arab populations because it shows how reference bias and ascertainment can distort genetic interpretation.

DCM also has acquired environmental contributors that can dominate the clinical phenotype: myocarditis, alcohol, chemotherapy, pregnancy, hypertension, ischemic disease, tachyarrhythmia, and metabolic disease. Reviews increasingly describe DCM architecture as complex, with oligogenic and modifier effects potentially explaining a meaningful fraction of disease [16]. Arrhythmogenic DCM represents another important layer, as variants in *LMNA*, *FLNC*, *DSP*, *RBM20*, and *PLN* may influence arrhythmia risk and management before severe systolic failure develops [17,18]. Precision imaging studies now attempt to connect genotype, chamber geometry, myocardial strain, and outcomes rather than relying on ejection fraction alone [19].

### 2.4. ACM Monogenic Architecture

ACM remains the cardiomyopathy subtype for which PRS evidence should be discussed with the greatest caution. The classical model is desmosomal disease involving *PKP2*, *DSP*, *DSG2*, *DSC2*, and *JUP*. Non-desmosomal genes such as *TMEM43*, *PLN*, *DES*, *LMNA*, and *FLNC* are clinically important but require careful phenotype-specific interpretation. A ClinGen-based reappraisal of *ARVC* genes strengthened the evidence base by separating definitive or strong associations from weaker historical claims [20].

Clinically, ACM is dominated by arrhythmic risk, exercise sensitivity, sudden cardiac death, and progressive structural disease. Reviews estimate prevalence around 1:2000 to 1:5000 and emphasize that most disease-causing variants occur in a limited set of desmosomal and related genes [21]. Left-dominant ACM and biventricular disease complicate the distinction from DCM [22]. Natural-history studies and variant-reassessment analyses show that both phenotype and genotype need re-evaluation over time [23,24]. In one ARVC cohort, left ventricular involvement emerged during follow-up; isolated right ventricular disease was present in 55%, biventricular disease in 19%, and new left ventricular involvement in 26%, with competitive exercise associated with progression [25].

We can conclude that ACM PRS is not clinically ready. Current ACM risk prediction work is still centred on phenotype, genotype, exercise exposure, ventricular function, arrhythmic history, and validated or externally tested risk calculators [6,26,27,28,29]. PRS may eventually explain incomplete penetrance, exercise-related expressivity, arrhythmia propensity, and overlap between *DSP*/*FLNC*/*LMNA* disease and DCM. Still, the field does not yet have sufficient disease-specific, multi-ancestry, prospective evidence to support the use of PRS in clinical ACM care.

Phenotypic boundaries can also blur within a single pedigree. In a Kuwaiti family described by Ebrahim et al., the pathogenic *DES* p.R127P (c.380G>C) variant was associated with a mixed cardiac phenotype including DCM, ACM, atrial fibrillation/conduction abnormalities, and substantial cardiac morbidity and mortality [30]. This family-level variability is clinically important: the same pathogenic cytoskeletal variant can produce different structural and electrical phenotypes, so rigid diagnostic silos may obscure shared genetic causation. Broader *DES* cohorts similarly demonstrate substantial overlap among arrhythmogenic, dilated, biventricular, and conduction-system phenotypes [31,32].

### 2.5. Restrictive Cardiomyopathy Monogenic Architecture

Primary restrictive cardiomyopathy (RCM) is a rare but clinically severe inherited myocardial disease characterized by impaired ventricular filling and marked diastolic dysfunction, often with biatrial enlargement despite non-dilated ventricles. Its genetic architecture overlaps substantially with HCM and DCM rather than forming an isolated molecular category. Reported disease genes include sarcomeric genes such as *TNNI3*, *TNNT2*, *ACTC1*, *MYH7*, and *MYBPC3*, and cytoskeletal or structural genes including *DES*, *FLNC*, *BAG3*, and *LMNA* [33]. Most familial sarcomeric and cytoskeletal RCM is autosomal dominant, although de novo and autosomal recessive disease are documented, including recessive *DES*-associated RCM. The same gene can therefore produce restrictive, hypertrophic, dilated, or arrhythmogenic phenotypes depending on the variant, age, and modifying background [33].

For PRS research, RCM presents a particularly difficult setting: disease prevalence is low, diagnostic boundaries overlap with infiltrative and hypertrophic phenotypes, and disease-specific GWAS or externally validated PRS are lacking. The near-term priority is therefore accurate monogenic diagnosis, harmonized phenotyping, longitudinal family studies, and multi-centre aggregation rather than clinical deployment of a stand-alone RCM PRS.

### 2.6. Why Monogenic Testing Is Insufficient

Monogenic testing is insufficient for five reasons. First, many patients with convincing cardiomyopathy remain genotype-negative. Second, penetrance is not binary; age, sex, ancestry, blood pressure, obesity, exercise, pregnancy, alcohol, infection, and therapy exposure can modify expression. Third, the same variant can produce different phenotypes within one family. Fourth, VUS rates increase when ancestry-specific allele-frequency data are limited. Fifth, rare-variant interpretation depends on reference databases that remain biased toward European ancestry.

For Arab patients, the problem is amplified. If an allele is common in an Arab subpopulation but rare in gnomAD European samples, it may be overcalled as suspicious. Conversely, a truly pathogenic founder variant can be missed or underinterpreted if local clinical and segregation data are absent. Monogenic testing alone, therefore, cannot quantify background inherited susceptibility, resolve VUS interpretation, or provide calibrated population-specific risk. The major genes, modifiers, interpretation problems, and PRS implications are summarized in Table 1.

## 3. Polygenic Risk Scores in Cardiovascular Disease

### 3.1. What PRS Is

A PRS aggregates the weighted effects of many common variants across the genome to estimate inherited susceptibility to a trait or disease. Unlike a monogenic result, the score does not identify one causal familial mutation; it summarizes a distributed background of common-variant liability. Construction requires a defined discovery GWAS, allele harmonization, an LD-aware weighting method, a quality-controlled and usually imputed target dataset, and independent validation. Reporting standards therefore require transparent details on ancestry, discovery data, target cohort, variant harmonization, imputation, effect weights, score distribution, calibration, and clinical interpretation [7]. The Polygenic Score Catalog provides score definitions and metadata that improve reproducibility, but catalogue deposition is not equivalent to clinical validity [34].

Transferability is the decisive methodological issue. Methods for improving multi-ancestry prediction exist, but they cannot fully compensate for the absence of ancestry-specific discovery data [35]. In the UK Biobank, portability analysis of 245 scores across nine ancestry groups showed that performance can decline when scores are transferred away from the discovery ancestry [36]. Reviews of cross-population PRS methods emphasize that ancestry differences in allele frequency, linkage disequilibrium, causal variant tagging, effect-size heterogeneity, and environmental context all influence performance [37,38,39]. Large diverse GWAS can improve discovery power and fine-mapping, as shown in lipid traits. However, the lesson for cardiomyopathy is uncomfortable: the relevant Arab cardiomyopathy datasets remain too small [40].

### 3.2. From GWAS Summary Statistics to a Polygenic Risk Score

Variant selection begins before score calculation. Discovery summary statistics should come from a well-phenotyped GWAS with ancestry and sample overlap documented. Alleles are harmonized to a common genome build; strand-ambiguous or poorly imputed variants are removed or resolved; variants failing minor-allele-frequency, Hardy–Weinberg, call-rate, or imputation-quality thresholds are excluded; and effect alleles must be aligned consistently between discovery, linkage-disequilibrium (LD) reference, and target datasets. Target genotypes undergo sample and variant quality control, phasing, and genotype imputation against an appropriate reference panel. Imputation quality is not a cosmetic detail: a score can lose accuracy when the reference panel poorly represents the target ancestry, especially for variants whose tagging patterns differ across populations [7,37,38,39,41].

The score is then a weighted sum of risk alleles, but the method used to estimate or shrink those weights matters. Pruning/clumping-and-thresholding (P + T) retains approximately independent variants after LD clumping and selects them using one or more GWAS *p*-value thresholds. It is transparent and computationally simple but discards correlated information and requires threshold tuning. LDpred2 instead uses a Bayesian point-normal model with an LD matrix to estimate posterior SNP effects across the genome; grid and auto implementations differ in how model parameters are selected, and performance depends on the quality and ancestry match of the LD reference [42]. PRS-CS uses Bayesian regression with a continuous-shrinkage prior, jointly shrinking noisy effects while retaining genome-wide polygenic information; PRS-CS-auto estimates the global shrinkage parameter from the summary statistics, and PRS-CSx extends the framework across ancestry-specific GWAS and LD panels [43]. These methods are not interchangeable labels: they embody different assumptions about polygenicity, LD, sparsity, and the portability of effect estimates.

Model validation must be separated from model development. Hyperparameters are tuned only in a training or tuning set, after which the model is locked and evaluated in an independent cohort without discovery-sample overlap. Internal cross-validation is useful for model selection but does not replace external validation. Disease association should be reported as OR or HR per score standard deviation with confidence intervals; discrimination should include AUROC or C-statistic; calibration should compare predicted and observed risk; and incremental value should be tested beyond age, sex, family history, imaging, clinical risk factors, and monogenic variant status. Where management decisions are proposed, absolute risk, reclassification, and decision-curve analyses are more informative than a statistically significant association alone. Ancestry-specific validation is essential because a well-calibrated European score can still be systematically biased in Arab or other underrepresented populations [7,35,36,37,38,39].

### 3.3. PRS Is Not a Diagnostic Test by Itself

A PRS estimates probability, not causality. It cannot confirm HCM, DCM, ACM, LVNC, or restrictive cardiomyopathy. It cannot replace pathogenic variant testing, segregation analysis, cardiac imaging, ECG interpretation, family history, or clinical judgment. A high PRS may increase suspicion in a borderline phenotype, but it does not equal disease. A low PRS does not exclude monogenic disease, especially when a strong pathogenic variant or family history is present.

This distinction matters because PRS can appear numerically precise yet be clinically unvalidated. A change in percentile or standard deviation is not useful unless it changes management safely. In inherited cardiomyopathy, premature use could increase surveillance burden in healthy relatives, misclassify secondary hypertrophy as genetic disease, or falsely reassure rare-variant carriers with low common-variant burden.

### 3.4. Clinical Utility Criteria

A cardiomyopathy PRS should not enter routine care merely because it is statistically significant. It needs analytical validity, clinical validity, incremental value, actionability, equity, cost-effectiveness, and ethical acceptability. Analytical validity means reproducible genotyping, imputation, and score calculation. Clinical validity means prediction in external cohorts with well-defined phenotypes. Incremental value means improvement beyond existing clinical, imaging, monogenic, and family-history models. Actionability means that the result changes surveillance intervals, family screening, counseling, preventive management, or research stratification. Equity means acceptable performance across ancestries, not only in European biobanks. Cost-effectiveness and ethical acceptability determine whether implementation improves healthcare rather than adding expensive uncertainty.

The cardiovascular PRS implementation literature increasingly warns against a simplistic translation of associations into practice. Clinical consensus statements now emphasise calibration, absolute risk, intended-use population, health-system readiness, and decision impact [44,45,46,47,48,49]. Those cautions are even more important in inherited cardiomyopathy, where the clinical consequences of a genetic label extend to relatives.

### 3.5. PRS Performance Metrics and Validation Standards

**Figure 1:**
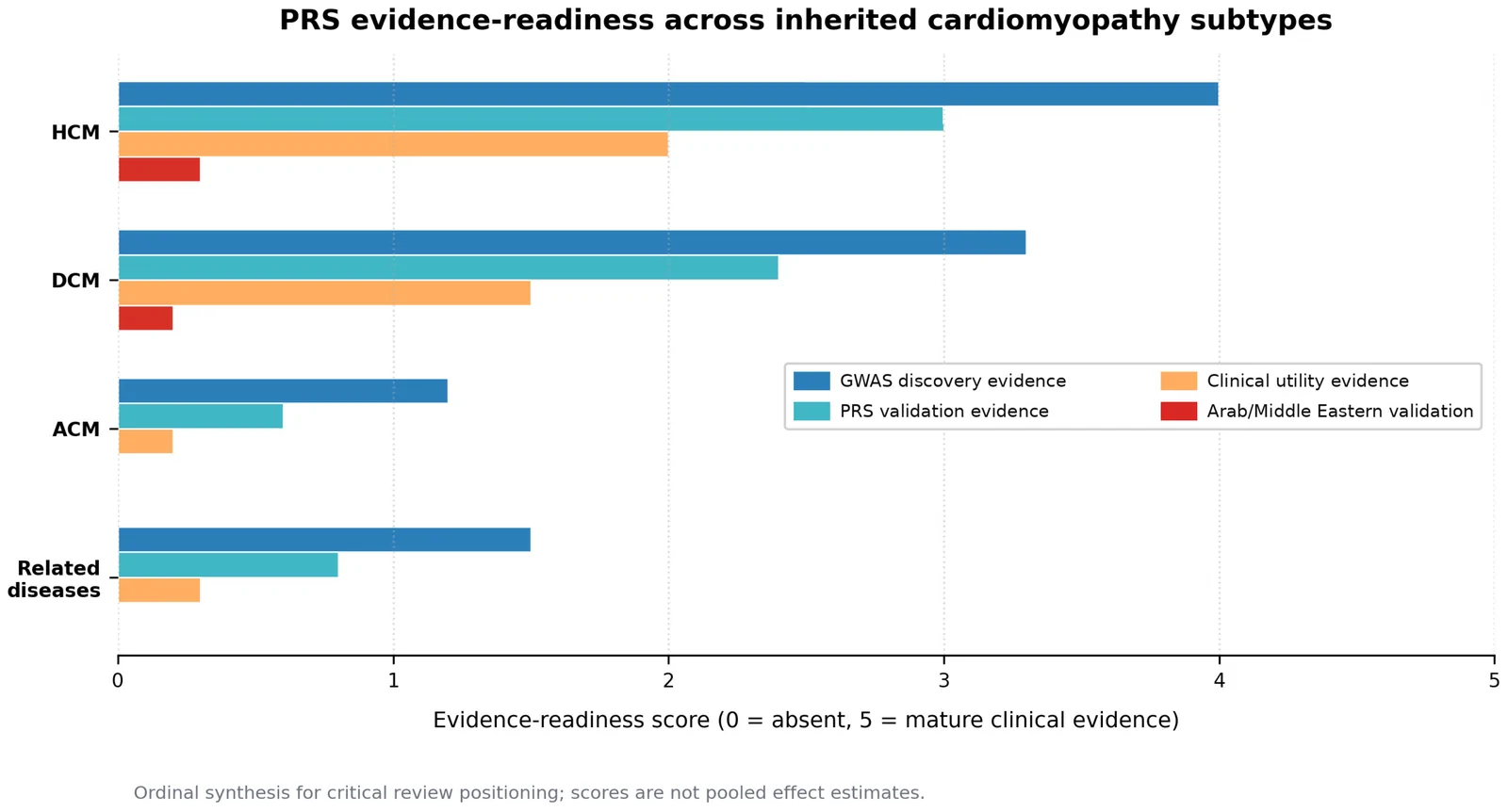
Evidence-readiness across inherited cardiomyopathy subtypes. This chart summarizes the relative maturity of PRS evidence by disease group and evidence domain. HCM has the strongest current PRS evidence; DCM is translational but more heterogeneous; ACM remains evidence-limited; and Arab/Middle Eastern validation is absent or very limited across all categories.

PRS studies should report odds ratios or hazard ratios per standard deviation, confidence intervals, area under the receiver-operating curve, C-statistic changes, net reclassification improvement, calibration slope, decision-curve analysis, absolute risk, relative risk, top-percentile risk, external validation performance, and ancestry-specific calibration. For family-based cardiomyopathy use, penetrance estimates by genotype and PRS stratum are more clinically meaningful than abstract discrimination metrics. A PRS with a modest increase in AUC may still be useful if it safely reclassifies relatives into different surveillance pathways; conversely, a statistically strong association may be useless if no management changes follow. Table 2 summarizes the utility domains and performance metrics reviewers should expect, and Figure 1 displays relative evidence-readiness across cardiomyopathy subtypes.

**Figure 2:**
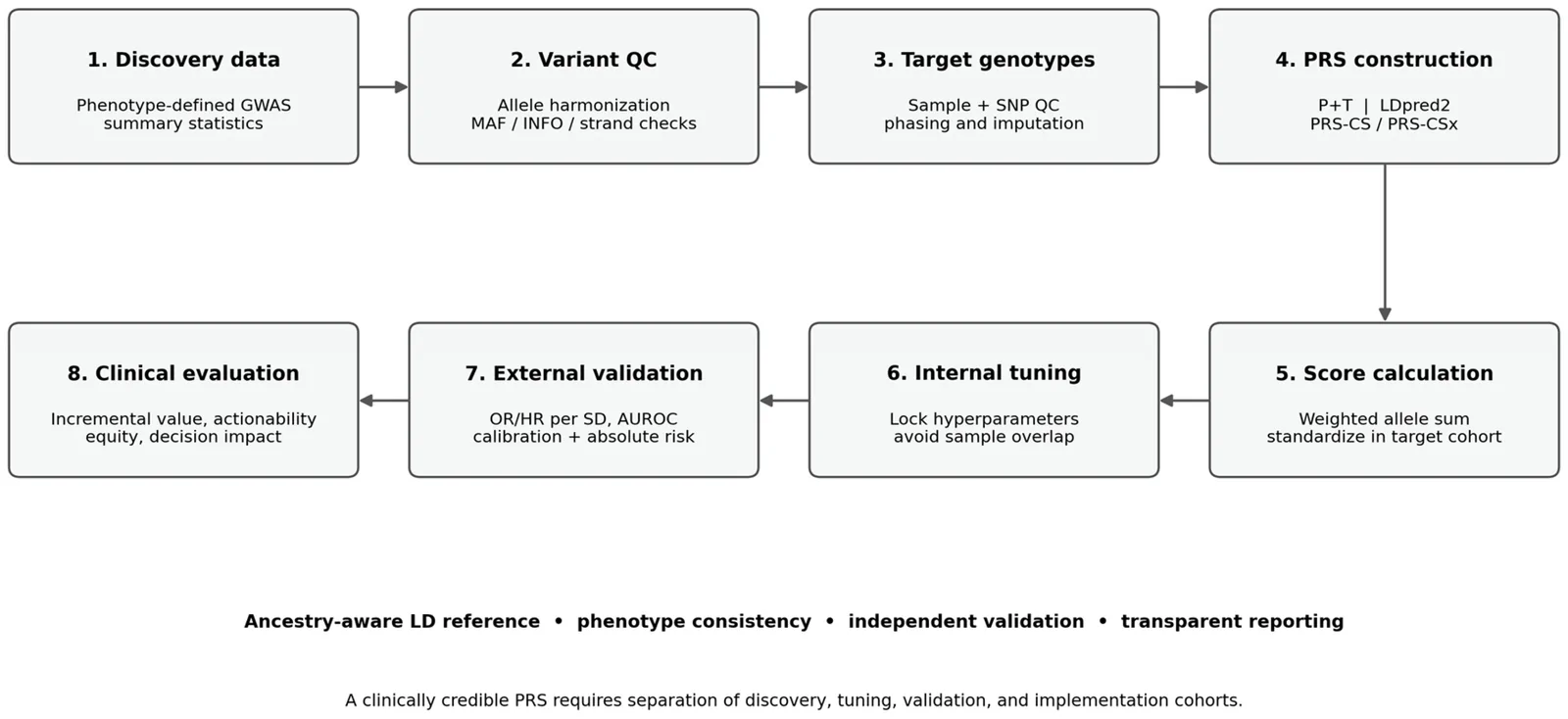
End-to-end development and clinical evaluation of a cardiomyopathy polygenic risk score. Discovery GWAS, variant harmonization, target-genotype quality control and imputation, LD-aware score construction, tuning, external validation, and clinical decision-impact assessment are deliberately separated to reduce overfitting and improve transportability.

Figure 2 summarizes the complete PRS development pathway, emphasizing the points at which ancestry mismatch, sample overlap, or inadequate validation can generate apparently strong but clinically misleading performance. Table 3 then compares major disease-specific cardiomyopathy scores using the reporting elements most relevant to reproducibility and translation.

## 4. PRS in HCM, DCM, ACM, and Related Disorders

### 4.1. PRS in HCM

HCM currently provides the strongest cardiomyopathy PRS model. In a landmark GWAS and modifier analysis, Harper and colleagues studied 2780 HCM cases and 47,486 controls, identified 12 susceptibility loci, and estimated SNP heritability at 0.34 ± 0.02. The genetic risk score approximately halved HCM odds in the lowest quintile and doubled odds in the highest quintile, and it also modified disease severity among rare sarcomere-variant carriers [54]. This is the clearest demonstration that common variant burden is not simply noise around a monogenic signal; it can shape penetrance and expressivity.

Tadros and colleagues showed that shared genetic pathways contribute to the risk of HCM and DCM in opposite directions, linking cardiomyopathy risk to quantitative ventricular traits [55]. Biddinger and colleagues then tested rare and common variation in 184,511 UK Biobank participants. Among 204 HCM cases, pathogenic or likely pathogenic variants in 14 core *HCM* genes were associated with an odds ratio of 55 (95% CI, 35–83), whereas the polygenic score was associated with an odds ratio of 1.6 per standard deviation (95% CI, 1.4–1.8). Importantly, adding rare variants and the polygenic score to clinical risk factors improved AUC from 0.71 to 0.82 [50]. That is incremental value, although still not proof of clinical utility.

The evidence has expanded rapidly. A 2025 large-scale HCM GWAS reported 5900 cases, 68,359 controls, 36,083 UK Biobank CMR studies, 70 HCM loci, and 62 left-ventricular trait loci, strengthening the mechanistic bridge between common variants and hypertrophic remodeling [56]. Another 2025 evaluation reported that polygenic scores stratified HCM risk across population and clinical settings and that penetrance among pathogenic-variant carriers differed by roughly 10-fold between the highest and lowest polygenic score quintiles [51]. Imaging-derived phenotype GWAS also supports the biological plausibility of PRS by identifying 72 loci associated with regional left ventricular wall thickness in 42,194 UK Biobank CMR studies and showing causal links to HCM traits at *p* < 0.01 [57]. A 2026 biobank analysis further found that monogenic and polygenic risk independently modified HCM and DCM status, as well as echocardiographic measures, in 49,434 participants [58].

The clinically relevant use of HCM PRS is probably not population screening. The stronger use case is risk refinement among relatives, genotype-positive/phenotype-negative individuals, sarcomere-negative HCM, and borderline hypertrophy. PRS may help distinguish monogenic HCM from polygenic or secondary left ventricular hypertrophy, but only if models are calibrated to ancestry and adjusted for hypertension, obesity, sex, age, and imaging definitions. A European-derived PRS applied uncritically to Arab patients with hypertension-associated LVH could convert uncertainty into false confidence.

### 4.2. PRS in DCM

DCM PRS evidence is growing stronger, but clinical implementation is more challenging. Zheng and colleagues reported a large DCM GWAS integrating 14,256 DCM cases and 36,203 UK Biobank CMR studies, identifying 80 loci and 62 candidate effector genes [52]. Jurgens and colleagues separately analysed 9365 DCM cases and 946,368 controls, identifying 70 genome-wide significant loci and 63 prioritized genes, and framed DCM risk partly around myocardial resilience [53]. These studies move DCM beyond single-gene interpretation, but they also show that DCM PRS may capture broad myocardial vulnerability rather than disease-specific causality.

The DCM PRS literature now argues for translation, but still mainly as a research-to-clinic pathway [59]. A clinically useful DCM PRS would need to explain why some *TTN* truncating-variant carriers develop DCM while others do not; why some individuals exposed to alcohol, pregnancy, or chemotherapy develop cardiomyopathy; and why idiopathic DCM clusters in families without clear monogenic findings. The strongest near-term use is not a standalone diagnosis; it is integration with *TTN* status, *LMNA*/*FLNC*/*DSP*/*RBM20*/*PLN* arrhythmogenic risk, CMR phenotype, ejection fraction trajectory, and exposure history.

Recent evidence supports this vulnerability model. In 2025, a study of peripartum, alcohol-induced, and cancer therapy-related cardiomyopathies reported that individuals with high DCM polygenic scores had approximately threefold increased odds of these acquired or exposure-associated cardiomyopathies [60]. That result is important because it undermines the clean boundary between genetic and acquired DCM. It also creates an implementation problem: if PRS predicts susceptibility across multiple cardiomyopathy triggers, clinicians need exposure-specific risk models rather than a single DCM label. Related imaging and electrophysiologic trait studies, including trabeculation architecture and resting heart rate PRS, further link common-variant burden to myocardial morphology and arrhythmic physiology [61,62]. Emerging work linking cardiomyopathy gene variants and PRS to atrial fibrillation also suggests that common and rare risk may interact across inherited myocardial and arrhythmia phenotypes [63].

DCM PRS is therefore promising but less clean than HCM PRS. Phenotype misclassification, heart failure overlap, acquired causes, inconsistent imaging definitions, and insufficient non-European validation all weaken immediate clinical readiness. The right conclusion is not that DCM PRS is useless. The right conclusion is that its clinical value will depend on separating genetic myocardial susceptibility from heterogeneous final common pathways of systolic failure.

### 4.3. PRS in ACM

For ACM, PRS is a research priority, not a clinical tool. Current evidence remains dominated by rare desmosomal and arrhythmogenic variants, phenotype criteria, exercise exposure, ventricular function, arrhythmic history, and family history. PRS may eventually help explain incomplete penetrance, left-dominant ACM, heterogeneity in arrhythmic risk, and overlap with *DCM* genes such as *DSP*, *FLNC*, and *LMNA*. However, small cohorts, phenotype evolution, diagnostic uncertainty, and the high clinical consequence of sudden-death risk make premature PRS implementation unsafe.

The more defensible research strategy is to build ACM registries with standardized diagnostic criteria, exercise-exposure measurement, CMR, Holter monitoring, ICD therapy outcomes, and longitudinal follow-up, and then test whether common variant burden adds value beyond known risk calculators. Until that exists, ACM PRS should be described as hypothesis-generating. Exaggerating ACM PRS evidence would weaken the review and invite reviewer criticism.

### 4.4. Related Inherited Cardiac Diseases and Traits

**Figure 3:**
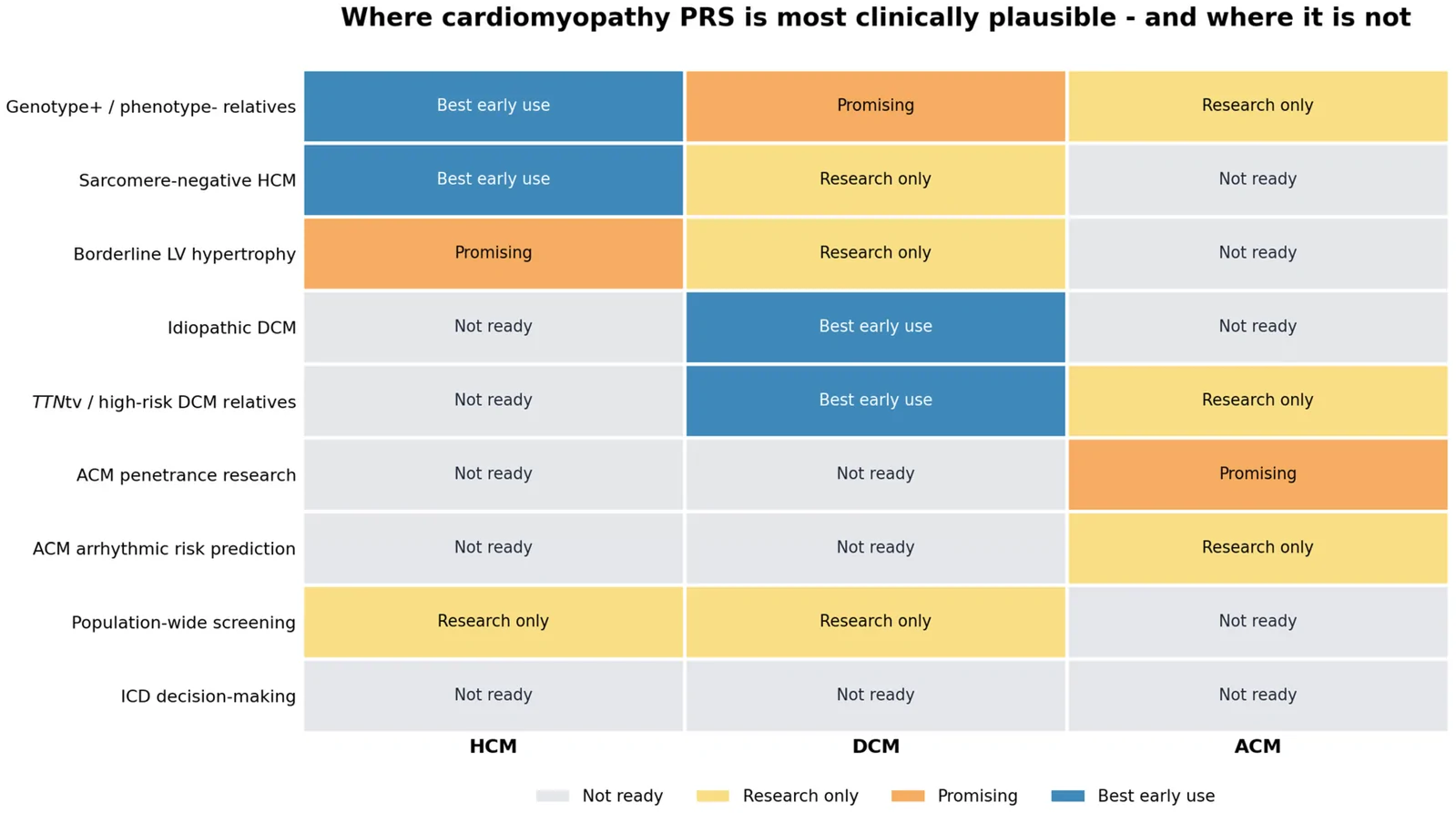
Clinical-use readiness matrix for cardiomyopathy PRS. The matrix identifies where PRS is most plausibly useful and where it should not be overclaimed. The most defensible early applications are risk refinement in HCM relatives, sarcomere-negative or borderline HCM, idiopathic DCM, and rare-variant DCM relatives. ACM, ICD decisions, and population-wide screening remain insufficiently supported.

PRS evidence is still limited for LVNC, restrictive cardiomyopathy, and arrhythmia-overlap syndromes. For RCM specifically, the problem is not merely a shortage of scores: the available cohorts are small, molecular causes overlap with HCM and DCM, and restrictive physiology can arise from non-genetic or syndromic disease. Current evidence therefore supports monogenic testing and family-based phenotyping, not a disease-specific RCM PRS [33]. The most useful adjacent polygenic evidence comes from cardiac MRI traits, myocardial trabeculation, atrial fibrillation, resting heart rate, ventricular dimensions, and heart failure traits. These studies map common-variant effects onto cardiac structure and function but should not be treated as validation of an RCM- or LVNC-specific score. Harmonized definitions and careful separation from normal morphologic variation or phenocopies are prerequisites before PRS can become clinically meaningful. The comparative evidence across cardiomyopathy subtypes is summarized in Table 4, and the likely clinical-use settings are mapped in Figure 3.

## 5. Challenges and Limitations

### 5.1. VUS and Variant Reinterpretation

VUS is not a minor inconvenience; it is one of the major reasons ancestry-aware implementation is necessary. VUS interpretation depends on allele frequency, computational prediction, functional evidence, segregation, case enrichment, phenotype match, and gene validity. Underrepresented populations have higher uncertainty because benign ancestry-specific variants may be absent from reference databases. The consequence can be unnecessary anxiety, inappropriate cascade testing, over-surveillance, or false reassurance. Variant reinterpretation studies in arrhythmogenic disorders show that classifications can change as evidence accumulates, which means one-time genetic reports are not enough [64,65].

In Arab populations, the VUS problem intersects with consanguinity, founder effects, endogamy, and incomplete regional reference data. Consanguinity increases the visibility of rare recessive and homozygous variants, but it does not automatically improve PRS performance. PRS is largely built from common-variant effect sizes and LD patterns; family-based rare-variant enrichment is a different problem.

### 5.2. Ancestry Bias and PRS Portability

European discovery bias is the central threat to PRS transferability. Differences in allele frequencies, LD, causal variant tagging, imputation quality, gene-environment correlation, and clinical ascertainment all affect calibration. A score can preserve rank ordering but still misestimate absolute risk. That is dangerous in inherited cardiomyopathy because clinical decisions involve relatives, surveillance, and sometimes device discussions. Arab populations are internally structured; North African, Levantine, Gulf, Arabian Peninsula, and admixed diaspora populations cannot be treated as genetically identical. A single Arab PRS may be too simplistic unless it is built and calibrated with substructure in mind.

### 5.3. Population Stratification and Technical Limitations

Population stratification can create false associations when cases and controls differ by ancestry rather than disease biology. Within-Arab substructure, founder effects, migration history, and variable admixture can distort both rare variant interpretation and GWAS. Technical limitations include small sample sizes in cardiomyopathy GWAS, phenotypic heterogeneity, genotyping arrays not optimized for Arab populations, non-Arab imputation panels, limited development of WGS-based PRS, and uncertain clinical thresholds. These are not cosmetic limitations. They determine whether a PRS is calibrated enough to inform clinical care.

### 5.4. Clinical, Ethical, and Policy Limitations

**Figure 4:**
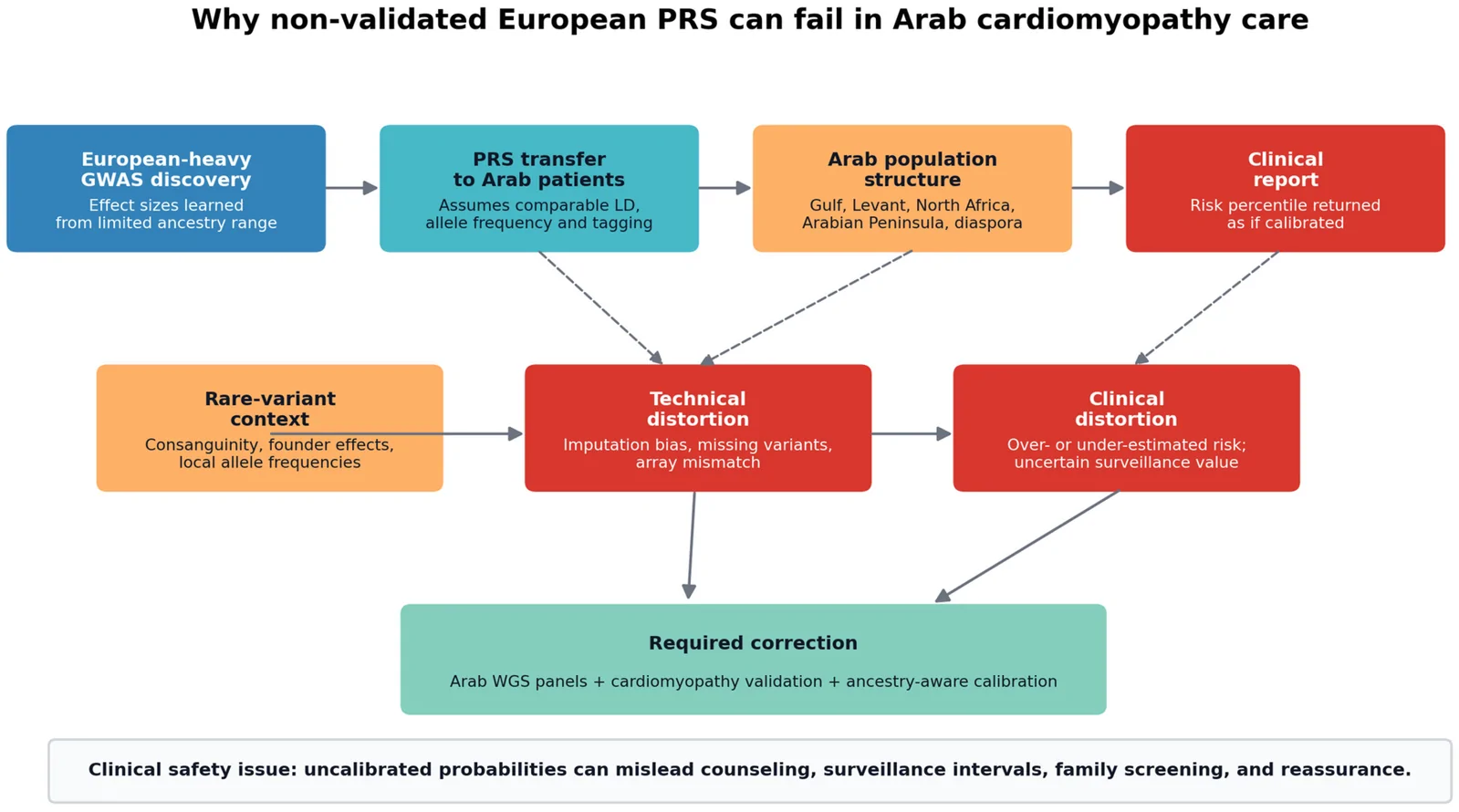
Mechanisms of ancestry portability failure in Arab cardiomyopathy PRS. This causal chart shows why PRS portability problems are clinically important. Differences in linkage disequilibrium, allele frequencies, population structure, founder effects, imputation performance, and calibration can convert a statistically valid score in one ancestry into a misleading clinical report in another.

No inherited cardiomyopathy subtype has a universally accepted PRS cutoff for clinical care. Prospective data are limited, and it remains unclear whether PRS should alter ICD decisions, exercise advice, pregnancy counseling, family screening intervals, or CMR frequency. Clinician literacy and counseling capacity are also limited. Ethically, PRS introduces probabilistic risk, family implications, privacy concerns, fears of insurance and employment discrimination, commercial misuse, and cross-border data governance challenges. In Arab societies, culturally appropriate counseling must address family communication, consanguinity, reproductive decision-making, and privacy without stigmatizing patients or families. The mechanisms through which ancestry portability can fail are illustrated in Figure 4.

## 6. Arab and Middle Eastern Populations: The Missing Piece

### 6.1. Why Arab Populations Matter

Arab and Middle Eastern populations matter scientifically and clinically because they are large, young in many countries, cardiometabolically burdened, and increasingly engaged in national genomic medicine programs. The region includes high rates of consanguinity in some communities, founder variants, extended family structures, and family-based disease clustering. These features create opportunities for variant discovery and penetrance studies, but only if the research is locally led, ethically governed, and connected to clinical outcomes.

Regional genomic medicine reviews argue that the Middle East can contribute substantially to human genetics research due to its population structure, rare disease burden, and expanding sequencing capacity [66,67]. However, national genome programs alone do not solve the problem of cardiomyopathy PRS. Population WGS can improve reference panels and variant frequencies, but PRS implementation also needs disease-specific GWAS, echo/CMR linkage, longitudinal outcomes, and family-based penetrance data.

### 6.2. Why Arab PRS Cannot Simply Be Copied from European Models

Directly copying European PRS into Arab cardiomyopathy care is scientifically weak. LD differences mean that European tag variants may not capture the same causal architecture. Allele-frequency differences can shift score distributions. Missing Arab-specific variants and imputation errors can reduce performance. Phenotype coding may differ across healthcare systems, and environmental modifiers such as obesity, hypertension, diabetes, exercise, family size, and access to advanced imaging may affect observed penetrance. Without Arab cardiomyopathy-specific validation cohorts, a European PRS can be statistically elegant and clinically misleading.

### 6.3. Current Arab Genomic Resources and What Remains Missing

The region now has important resources. Reviews of MENA population genome programs describe national and regional efforts in Qatar, Saudi Arabia, the United Arab Emirates, and other regions [68]. The Qatar Genome Program reported actionable genomic variants in 6045 participants, identifying 60 pathogenic or likely pathogenic variants in 25 *ACMG* genes among 141 unique individuals [69]. Qatar Genome analyses have also provided broader insight into Middle Eastern genomic architecture [70], and a 2024 analysis of 14,392 whole genomes reported that approximately 3.5% of Qataris carried clinically actionable findings [71]. The UAE has developed a population-specific major-allele reference genome using whole-genome and whole-exome data, reporting millions of short and structural variants, including novel variants [72]. Saudi sequencing studies have estimated secondary genomic findings in local populations, including an 8% rate in one analysis of 1254 individuals [73].

These resources are valuable but incomplete for inherited cardiomyopathy PRS. They do not replace HCM, DCM, or ACM GWAS. They do not automatically provide PRS validation, longitudinal cardiac imaging, family-based penetrance, ECG/echo/CMR linkage, ICD therapy outcomes, transplant outcomes, or sudden-death ascertainment. Saudi and regional reviews highlight the broader inherited-disease opportunity but also expose the need for structured databases and clinical translation pathways [74]. Public willingness data from Qatar show that population engagement is possible, but willingness is not equivalent to informed consent for family-based PRS use [75]. Near-complete Middle Eastern genome resources can refine reference genomes and variant discovery, but clinical cardiomyopathy implementation still requires disease-specific cohorts [76]. Consanguineous cardiomyopathy family studies illustrate the power of family design for rare-variant discovery, yet this design addresses a different question than population PRS calibration [77].

### 6.4. Specific Arab Cardiomyopathy Research Gaps

**Figure 5:**
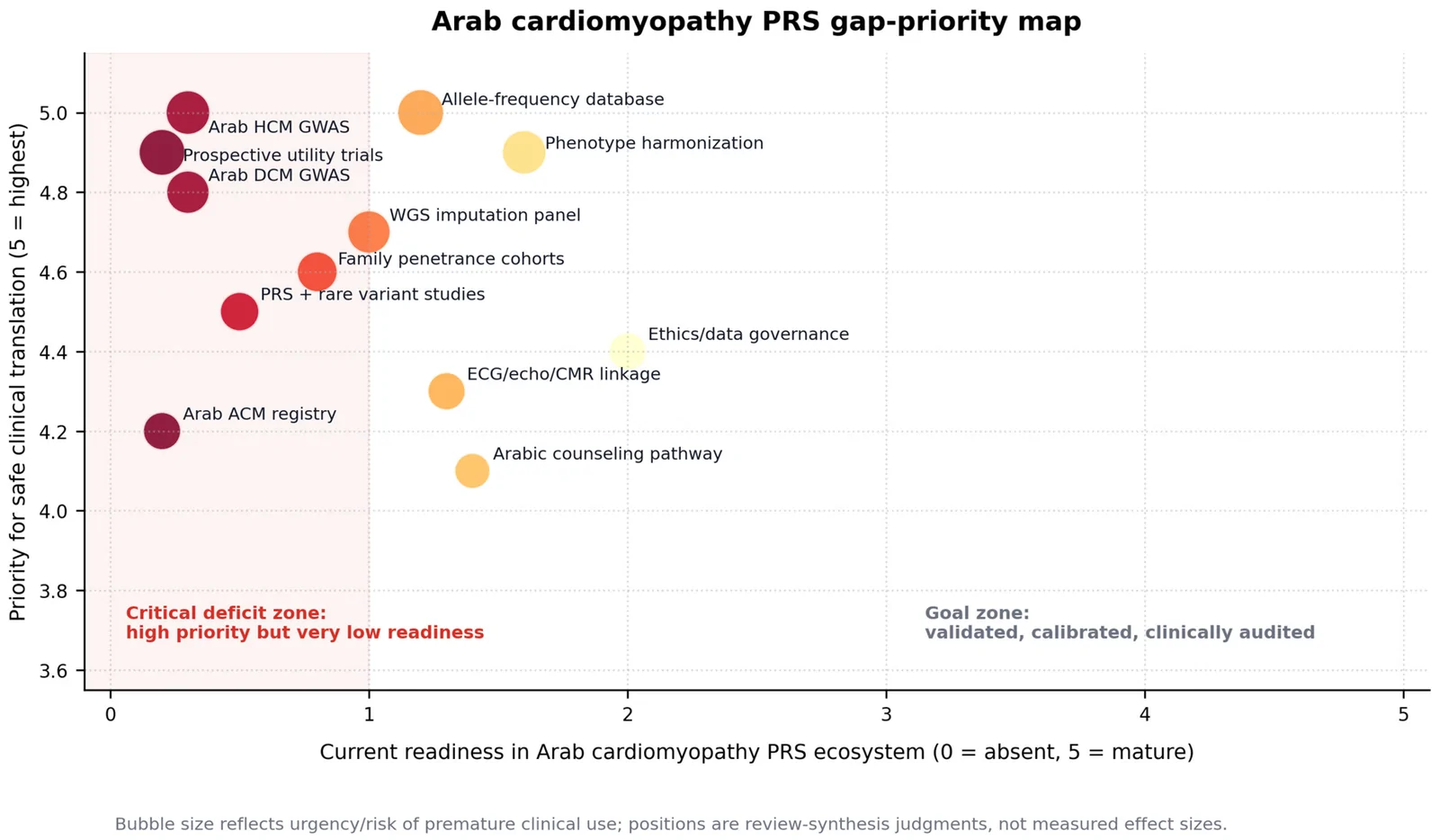
Arab cardiomyopathy PRS gap-priority map. The map separates urgency from readiness. The highest-risk deficits are not merely academic gaps; they are the areas most likely to lead to incorrect risk estimates, VUS misclassification, or premature clinical use when European-derived scores are imported without local validation. Colors indicate relative readiness: dark red marks the least developed, highest-priority gaps; orange indicates intermediate readiness; and yellow indicates greater relative readiness.

The research gaps are concrete: no adequately powered Arab HCM GWAS; no adequately powered Arab DCM GWAS; no Arab ACM GWAS; no validated Arab cardiomyopathy PRS; limited ancestry-specific allele-frequency panels for variant interpretation; limited VUS reclassification studies; limited family-based penetrance models; almost no PRS-plus-monogenic interaction studies; no prospective clinical utility trials; no economic evaluations; insufficient genetic counseling implementation research; and no Arab-specific PRS reporting standard. These gaps should be stated plainly. These gaps and required solutions are summarized in Table 5 and prioritized visually in Figure 5.

## 7. Clinical Implementation and Future Directions

### 7.1. Where PRS May Realistically Help

The realistic early use of PRS in inherited cardiomyopathy is risk refinement, not diagnosis. It may help genotype-positive/phenotype-negative relatives, sarcomere-negative HCM, borderline LV hypertrophy, idiopathic DCM, *TTN* variant carriers, families with variable penetrance, and research stratification for trials. It may eventually inform surveillance intensity if a validated model shows that high-PRS relatives develop disease earlier or that low-PRS relatives can safely avoid excessive imaging.

PRS is not ready for standalone diagnosis, ICD decisions, population-wide Arab screening, replacing cascade testing, or ruling out inherited disease. A low PRS must never override a pathogenic LMNA variant, a malignant family history, or clear imaging disease. A high PRS must never turn mild hypertensive remodeling into a definitive inherited HCM diagnosis.

### 7.2. Proposed Clinical Workflow

A responsible workflow starts with clinical phenotype confirmation, three-generation family history, ECG, echocardiography, CMR where indicated, and monogenic panel testing using genes with strong evidence. Variant classification should use ancestry-aware databases and segregation when possible. PRS should be considered only when validated for patient ancestry and intended clinical use. The result should feed into an integrated risk model, be discussed through genetic counseling, and generate a documented follow-up plan. Registry entry and outcome tracking should be mandatory in early implementation so the system learns whether PRS changes care safely.

### 7.3. Future Research Priorities

**Figure 6:**
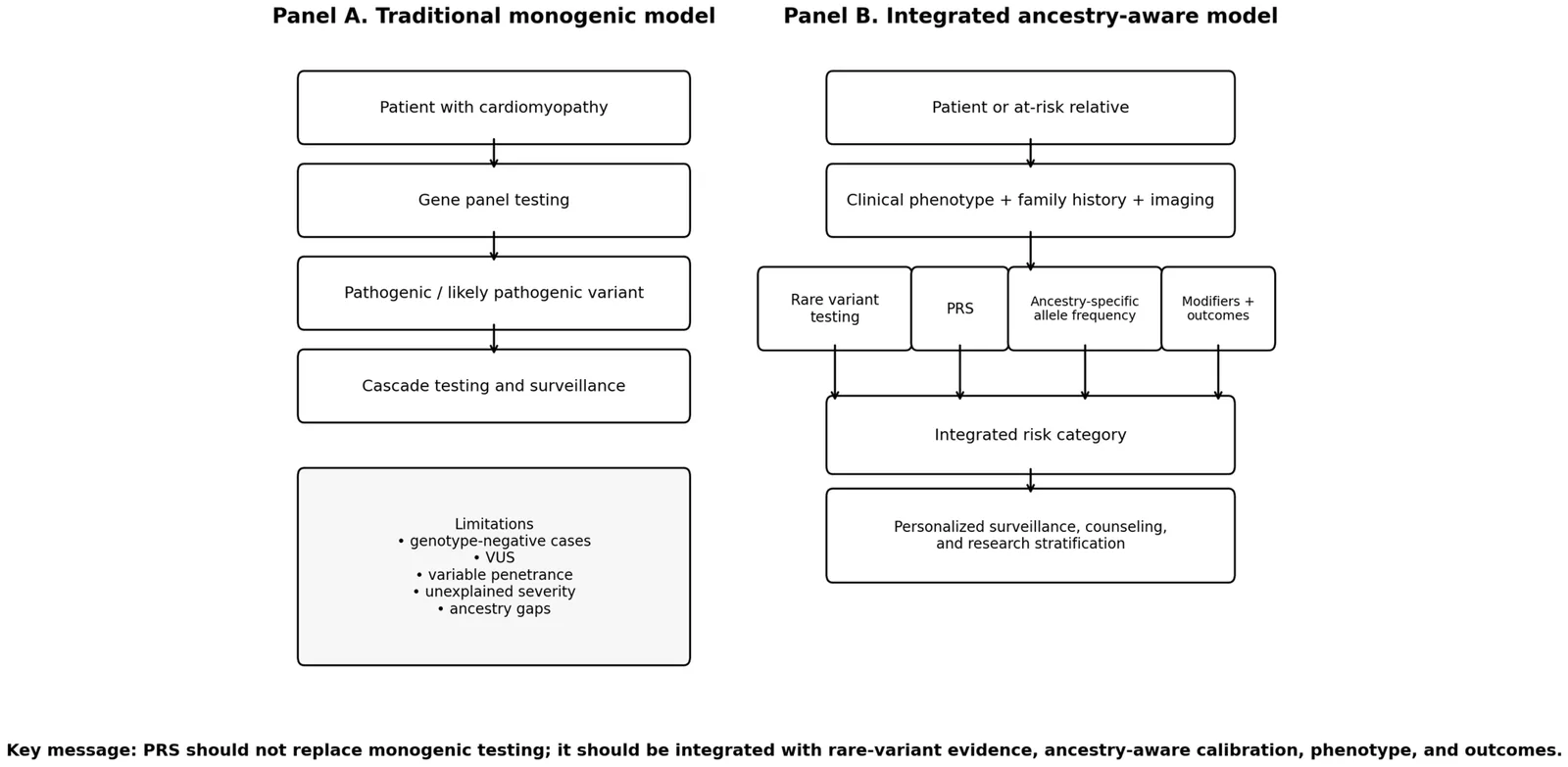
Transition from monogenic to integrated genomic risk assessment in inherited cardiomyopathies.

The research priorities are an Arab cardiomyopathy registry, harmonized phenotype definitions, WGS rather than array-only genotyping, ancestry-specific imputation panels, Arab HCM and DCM GWAS, ACM genotype–phenotype registries, trans-ethnic meta-analysis, PRS recalibration, family-based penetrance studies, integration with CMR and ECG, prospective clinical utility trials, health-economic evaluation, and an ethical governance framework. The priority is not to buy or license a commercial European PRS. The priority is to build evidence that can survive clinical audit. This research direction is summarized schematically in Figure 6.

Traditional inherited cardiomyopathy care moves from clinical diagnosis to gene panel testing, pathogenic or likely pathogenic variant identification, cascade testing, and surveillance. The integrated model retains monogenic testing but adds PRS, ancestry-specific allele frequencies, environmental modifiers, family history, imaging, and longitudinal outcomes. Key message: the future model should not replace monogenic testing with PRS; it should combine rare- and common-variant risk within ancestry-aware clinical frameworks.

## 8. Proposed Framework for Arab Populations

### 8.1. Phase 1: Discovery Infrastructure

The first phase is infrastructure, not PRS calculation. Arab centres should build a multicentre cardiomyopathy consortium with standardized diagnostic definitions for HCM, DCM, ACM, LVNC, and restrictive cardiomyopathy. Each participant should undergo deep phenotyping, including ECG, echocardiography, CMR, Holter or device data, exercise history, medication exposures, cardiometabolic traits, family pedigree, and longitudinal outcomes. WGS should be preferred over genotyping arrays because arrays miss local and structural variation. Ancestry mapping and local allele-frequency databases must be built into the design from the beginning.

### 8.2. Phase 2: PRS Development

The second phase is PRS development. Arab-specific GWAS should be performed where sample size allows, and trans-ethnic meta-analysis should be used to improve power without erasing local structure. PRS should be constructed using multiple methods, including LD-aware and multi-ancestry approaches, then compared transparently. LD reference panels should be derived from Arab WGS. Internal validation must be followed by external validation across Gulf, Levantine, North African, Arabian Peninsula, and diaspora Arab groups. This step is essential because a single regional average can hide subgroup miscalibration.

### 8.3. Phase 3: Clinical Validation

The third phase is clinical validation. PRS performance should be tested separately in HCM, DCM, ACM, genotype-positive relatives, genotype-negative probands, and borderline phenotypes. Outcomes should include disease onset, maximal LV wall thickness, LV dilation, ejection fraction decline, ventricular arrhythmia, sudden cardiac death, ICD therapy, heart failure admission, transplant, and mortality. Calibration, absolute risk, and family-based penetrance are more useful than association alone. A model that predicts coded disease in a biobank but fails in family clinics is not clinically useful.

### 8.4. Phase 4: Clinical Implementation

The fourth phase is controlled implementation. It requires genetic counseling pathways, clinician education, electronic health record integration, decision-support tools, patient-facing reports in Arabic and English, data governance policies, reimbursement models, and audits of downstream outcomes. Reports should avoid deterministic language. They should explain what the PRS can and cannot do, whether it was validated in patient ancestry, and how it changes management if at all.

### 8.5. Phase 5: Equity and Governance

**Figure 7:**
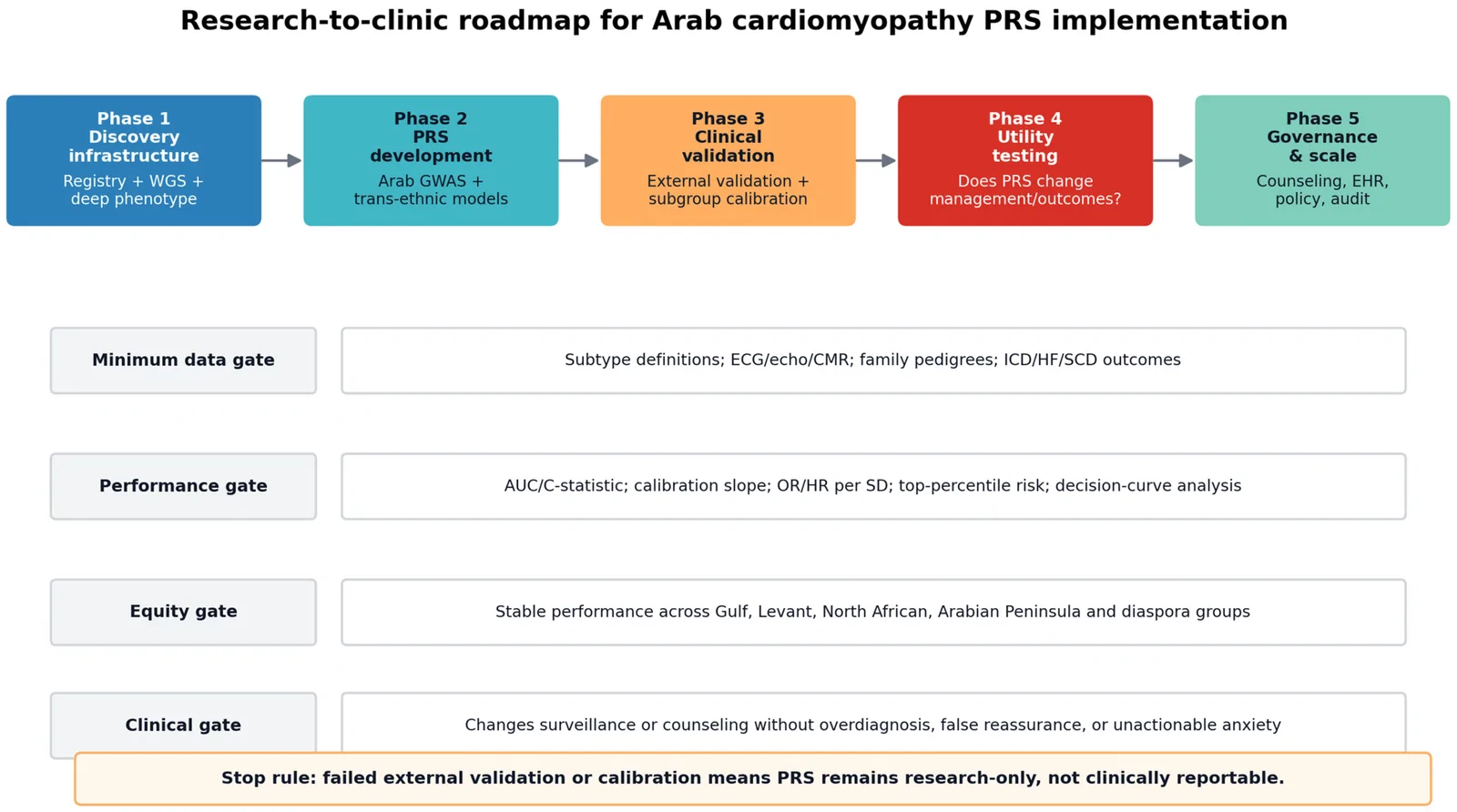
Research-to-clinic roadmap for Arab cardiomyopathy PRS implementation. Roadmap converts the proposed Arab framework into explicit go/no-go gates. It emphasizes that discovery infrastructure is not enough: clinical reporting should require external validation, ancestry-specific calibration, equity testing, and prospective evidence that the score changes management or improves outcomes.

The fifth phase is equity and governance. Community engagement, consent for family implications, privacy protection, anti-discrimination safeguards, cross-border data-sharing agreements, local scientific leadership, and transparent benefit-sharing are not optional. Arab genomics should not become extractive international genomics in which local populations provide data but do not control priorities, analysis, counseling models, or clinical implementation. Equity must be measured by performance, access, interpretation quality, and harm reduction. The phased implementation roadmap is summarized in Table 6 and translated into go/no-go gates in Figure 7.

## 9. Conclusions

Monogenic testing remains the foundation of inherited cardiomyopathy care. It identifies actionable pathogenic variants, enables cascade testing, and supports gene-informed management. But monogenic testing does not fully explain penetrance, expressivity, genotype-negative disease, or variable severity. PRS adds a second layer of inherited risk that is biologically and clinically plausible, especially in HCM and increasingly in DCM. ACM remains underdeveloped and should be treated as a research frontier rather than a clinical PRS application.

For Arab populations, the immediate priority is not clinical importation of European PRS. The priority is local discovery, WGS-based reference panels, cardiomyopathy-specific GWAS, ancestry-aware calibration, family-based penetrance studies, longitudinal ECG/echo/CMR outcome linkage, clinical utility trials, economic evaluation, counseling pathways, and governance. The future of inherited cardiomyopathy genetics is not monogenic versus polygenic. It is an integrated genomic risk, implemented only when it improves decisions safely, equitably, and measurably.

## Figures and Tables

**Table 1:** Monogenic architecture and modifiers across inherited cardiomyopathies.

Subtype	Core Genes (References)	Inheritance/Architecture	Modifiers	Interpretation Challenge	PRS Implication
HCM	*MYH7* [8], *MYBPC3* [8], *TNNT2* [8], *TNNI3* [8], *TPM1* [8], *ACTC1* [8], *MYL2* [8], *MYL3* [8]	Predominantly autosomal dominant (AD); incomplete and age-dependent penetrance; rare recessive/digenic forms	Age, sex, hypertension, obesity, exercise, metabolic traits, ancestry	Sarcomere-positive and sarcomere-negative HCM differ; borderline LVH can be overdiagnosed	Best current setting for rare-common variant integration
DCM	*TTN* [13,14], *LMNA* [13,14], *FLNC* [13,14], *DSP* [13,14], *RBM20* [13,14], *BAG3* [13,14], *PLN* [13,14], *DES* [13,31,32], *MYH7* [13,14], *TNNT2* [13,14]	Predominantly AD for major adult genes; autosomal recessive (AR), X-linked, mitochondrial, and syndromic forms also occur; oligogenic contribution possible	Alcohol, myocarditis, chemotherapy, pregnancy, hypertension, ischemia, tachyarrhythmia	Inherited vulnerability overlaps with acquired cardiomyopathy and HF phenotypes	PRS may capture myocardial vulnerability but needs phenotype-specific validation
ACM	*PKP2* [20], *DSP* [20], *DSG2* [20], *DSC2* [20], *JUP* [20], *TMEM43* [20], *PLN* [20], *DES* [20,31,32], *LMNA* [20], *FLNC* [20]	Usually AD with incomplete penetrance; AR desmosomal/syndromic forms are recognized; right-, left-, or biventricular disease	Exercise intensity, sex, variant class, ventricular involvement, arrhythmic history	Diagnostic overlap with DCM and myocarditis; high consequence of misclassification	PRS is a research priority, not a clinical tool
RCM	*TNNI3* [33], *TNNT2* [33], *ACTC1* [33], *MYH7* [33], *MYBPC3* [33], *DES* [31,33], *FLNC* [33], *BAG3* [33], *LMNA* [33]	Mostly AD for sarcomeric/cytoskeletal forms; de novo and AR disease also described	Age, variant class, myocardial fibrosis, conduction disease, systemic/phenocopy conditions	Strong overlap with HCM/DCM; small cohorts and etiologic heterogeneity complicate classification	No validated disease-specific PRS; multi-centre phenotype harmonization is prerequisite
LVNC and other related diseases	*MYH7* [8], *ACTC1* [8], *TNNI3* [8], *TNNT2* [8], *LMNA* [13], *FLNC* [13], *TTR* [2] and syndrome-specific genes [2]	AD, AR, X-linked, mitochondrial, or syndromic depending on cause; phenotype definitions vary	Loading conditions, age, imaging method, arrhythmia burden, systemic disease	Small cohorts and inconsistent definitions weaken genomic discovery	Use PRS only as exploratory research until disease-specific evidence matures

**Table 2:** Clinical utility criteria and PRS performance metrics for inherited cardiomyopathy.

Domain	What Must Be Shown	Preferred Metrics	Minimum Evidence Standard	Main Failure Mode
Analytical validity	Reproducible genotyping, imputation, variant harmonization, and score calculation	Call rate, imputation quality, concordance, score reproducibility	Independent technical validation	Black-box score calculation or ancestry-mismatched imputation
Clinical validity	Prediction of disease onset, phenotype severity, or outcomes	OR/HR per SD, AUC/C-statistic, calibration slope, absolute risk	External validation in phenotype-confirmed cohorts	Association in biobank coding only
Incremental value	Improves existing clinical/genetic models	Delta C-statistic, NRI, IDI, likelihood ratio, decision curves	Comparison against family history, ECG, echo, CMR, monogenic results	PRS looks significant but adds no clinical information
Actionability	Changes surveillance, counseling, imaging frequency, trial stratification, or prevention	Management reclassification and outcome impact	Prospective implementation or decision-impact study	Prediction without a management consequence
Equity	Acceptable performance across Arab subgroups and other ancestries	Subgroup calibration, ancestry-specific OR/HR, misclassification rates	Multi-centre external validation	European model imported without calibration
Cost and ethics	Value, consent quality, privacy, anti-discrimination safeguards	Cost per useful reclassification, patient understanding, harm monitoring	Health-economic and implementation study	Commercial overuse and family anxiety

**Table 3:** Comparative characteristics of major cardiomyopathy polygenic scores and validation studies.

Disease/Study	Discovery Sample	Algorithm/SNP Count	Key Performance	Validation Cohort(s)	Non-European Validation	Critical Interpretation
HCM—Biddinger et al. 2022 [50]	UK Biobank analysis: 184,511 participants, 204 HCM cases; score derived from prior HCM GWAS	Published weighted HCM polygenic score; exact construction algorithm and SNP count were not fully specified in the main report	OR 1.6 per SD (95% CI 1.4–1.8). Adding rare variants + PGS to clinical factors increased AUC 0.71 to 0.82	UK Biobank; concordant replication in Mass General Brigham Biobank	Not specifically demonstrated	Important early proof of incremental prediction, but limited score-construction reporting constrains reproducibility
HCM—Zheng et al. 2025 [51]	5900 HCM cases + 68,359 controls (European ancestry); MTAG also used CMR traits in 36,083 UKB participants	PRS-CS auto; 376,730 SNPs (GWAS score) and 374,113 SNPs (MTAG score)	MTAG PGS OR 2.34 per SD (95% CI 2.12–2.59) in independent UKB; AUROC and Nagelkerke R^2^ assessed	343,182 unrelated white British UKB; 100,000 Genomes; Erasmus MC relatives; Royal Brompton/Harefield	Yes, but limited: South Asian/African UKB performance was weaker/underpowered; East Asian PRS-CSx pilot	Best developed HCM PGS framework; strong association and penetrance modification, but ancestry portability remains incomplete
DCM—Zheng et al. 2024 [52]	14,256 DCM cases in large GWAS; CMR integration in 36,203 UKB participants	PRS-CS; 541,841 SNP predictors	OR 1.76 per SD (95% CI 1.64–1.90); AUROC 0.71 in UKB	347,585 unrelated white British UKB participants; rare-variant carrier analyses	No dedicated non-European external validation in the primary PGS analysis	Shows clinically relevant common-variant burden and *TTN* penetrance modification, but validation was predominantly European
DCM—Jurgens et al. 2024 [53]	9365 strict DCM cases + 946,368 controls; MTAG and replication resources expanded discovery	PRS-CS auto; PGS Catalog scores contain ~1.04–1.10 million variants depending on leave-one-cohort-out model	All of Us: OR per SD 1.73 European, 1.61 African, 1.34 admixed-American; additional discrimination metrics reported in supplementary analyses	Amsterdam UMC, UK Biobank, and All of Us with discovery-cohort exclusions to reduce overlap	Yes: All of Us African and admixed-American strata, although ancestry-specific discovery remained limited	Strongest evidence that a DCM PGS retains signal across multiple ancestries, but calibration and effect magnitude vary
ACM—current evidence	No adequately powered disease-specific ACM GWAS supporting a validated clinical PRS	No validated disease-specific algorithm/SNP set	No reproducible disease-specific AUROC/C-statistic or OR-per-SD benchmark suitable for clinical use	No validated clinical PRS cohort	No	The absence of a mature ACM PRS is itself a key result; rare-variant, phenotype, exercise, and validated arrhythmic-risk models remain primary

**Table 4:** Evidence for polygenic risk scores in inherited cardiomyopathies.

Subtype	Main Monogenic Genes	PRS/GWAS Evidence Level	Main PRS Use Case	Evidence Strength	Clinical Readiness	Main Limitations	Arab/Middle Eastern Validation	Future Priority
HCM	*MYH7*, *MYBPC3*, *TNNT2*, *TNNI3*, *TPM1*, *ACTC1*	Strongest among inherited cardiomyopathies; multiple GWAS/PRS and imaging-trait studies	Penetrance modification, genotype-negative HCM, borderline LVH, family risk refinement	Moderate to strong	Promising but not routine	European bias, threshold uncertainty, unclear surveillance impact, hypertension/obesity confounding	Absent or very limited	Arab HCM GWAS; family-based validation; CMR-linked PRS calibration
DCM	*TTN*, *LMNA*, *FLNC*, *DSP*, *RBM20*, *BAG3*, *PLN*	Emerging strong GWAS evidence; PRS may capture myocardial vulnerability	*TTN* penetrance, idiopathic DCM, systolic dysfunction, exposure-associated susceptibility	Moderate	Research-to-translational	Acquired causes, HF overlap, heterogeneous definitions, unclear actionability	Absent or very limited	Arab DCM cohorts with WGS, imaging, exposure data, and longitudinal outcomes
ACM	*PKP2*, *DSP*, *DSG2*, *DSC2*, *JUP*, *TMEM43*, *PLN*	Limited disease-specific PRS evidence	Future penetrance, expressivity, and arrhythmia-risk research	Weak	Not ready	Rare disease, small cohorts, diagnostic evolution, high consequence of false reassurance	Absent	ACM registry with standardized phenotype, exercise, CMR, Holter, ICD outcomes
Related diseases	*LVNC*/*RCM*/arrhythmia-overlap genes vary by phenotype	Mostly trait-level evidence, not disease-specific validation	Research stratification and trait biology	Limited	Not ready	Small samples, inconsistent phenotype definitions, normal-variant overlap	Absent	Harmonized phenotyping and multi-ancestry genomic studies

**Table 5:** Genomic research gaps limiting PRS implementation in Arab inherited cardiomyopathy care.

Gap Domain	Current Problem	Why It Matters for PRS	Consequence for Arab Patients	Required Solution	Priority
GWAS representation	Arab populations underrepresented in cardiomyopathy GWAS	European effect sizes and LD tags may not transfer	Inaccurate risk estimates and poor calibration	Arab HCM/DCM/ACM GWAS and trans-ethnic meta-analysis	Very high
Variant databases	Limited Arab allele-frequency and segregation data	Rare variant and PRS interpretation both require local background frequencies	False pathogenicity, missed founder variants, high VUS burden	Arab reference genomes, variant databases, and disease registries	Very high
Phenotype harmonization	Inconsistent HCM/DCM/ACM definitions and imaging protocols	GWAS signal quality depends on phenotype accuracy	Weak models and misleading associations	Standardized ECG, echo, CMR, Holter, ICD and outcome definitions	Very high
Family penetrance	Limited longitudinal Arab family studies	PRS must be tested in relatives and carriers, not only cases	Unclear counseling value and surveillance intensity	Family registries with cascade testing and follow-up	High
Imputation panels	Non-Arab reference panels dominate imputation	Poor tagging of local variants reduces score accuracy	Reduced PRS performance and biased percentiles	WGS-based Arab imputation reference panels	High
Clinical utility	No prospective Arab PRS implementation trials	Prediction does not equal clinical benefit	Premature clinical use and wasted resources	Decision-impact, outcomes, and cost-effectiveness studies	Very high
Ethics/governance	Limited regional policy for PRS, family data, and cross-border sharing	Family risk data are sensitive and probabilistic	Anxiety, discrimination, privacy harms, commercial misuse	Consent, counseling, anti-discrimination and data-governance frameworks	High

**Table 6:** Proposed phased Arab implementation roadmap for cardiomyopathy PRS.

Phase	Core Aim	Required Data	Main Deliverables	Go/No-Go Criterion	Risk If Skipped
1. Discovery infrastructure	Build Arab cardiomyopathy consortium and phenotype registry	ECG, echo, CMR, Holter, WGS, pedigrees, exposures, outcomes	Standardized dataset and allele-frequency resource	Multi-centre recruitment and phenotype quality audit	PRS built on weak or biased phenotype labels
2. PRS development	Generate Arab and trans-ethnic scores	Arab GWAS, WGS LD panels, imputation resources	Candidate PRS models and transparent reporting	Internal validation and method comparison	European score imported without calibration
3. Clinical validation	Test prediction in real clinical subgroups	HCM/DCM/ACM cohorts, relatives, genotype-negative and borderline cases	Calibration, discrimination, absolute risk, penetrance estimates	External validation across Arab subgroups	Misclassification and false reassurance
4. Implementation	Integrate into counseling and follow-up pathways	EHR, reports, clinician training, genetic counseling capacity	Decision-support tools and audited management protocols	Evidence that PRS changes care safely	Expensive probabilistic result with no action
5. Equity/governance	Protect patients and ensure local benefit	Consent, privacy, data-sharing, patient engagement, policy	Anti-discrimination and governance framework	Community trust and accountable oversight	Commercial misuse, stigma, extraction, and inequity

## Data Availability

No new data were created or analyzed in this study. Data sharing is not applicable to this article.
